# Cross-sectional associations among P3NP, HtrA, Hsp70, Apelin and sarcopenia in Taiwanese population

**DOI:** 10.1186/s12877-021-02146-5

**Published:** 2021-03-20

**Authors:** Yuan-Yuei Chen, Yi-Lin Chiu, Tung-Wei Kao, Tao-Chun Peng, Hui-Fang Yang, Wei-Liang Chen

**Affiliations:** 1 Department of Pathology, Tri-Service General Hospital; and School of Medicine, National Defense Medical Center, Taipei, Taiwan, Republic of China; 2 Department of Pathology, Tri-Service General Hospital Songshan Branch; and School of Medicine, National Defense Medical Center, Taipei, Taiwan, Republic of China; 3 Division of Geriatric Medicine, Department of Family and Community Medicine, Tri-Service General Hospital; and School of Medicine, National Defense Medical Center, Number 325, Section 2, Chang-gong Rd, Nei-Hu District, 114 Taipei, Taiwan, Republic of China; 4 Department of Biochemistry, National Defense Medical Center, Taipei, Taiwan, Republic of China

**Keywords:** Gender, Sarcopenia, Inflammation, Muscle loss, Biomarkers

## Abstract

**Background:**

Sarcopenia is a multifactorial pathophysiologic condition of skeletal muscle mass and muscle strength associated with aging. However, biomarkers for predicting the occurrence of sarcopenia are rarely discussed in recent studies. The aim of the study was to elucidate the relationship between sarcopenia and several pertinent biomarkers.

**Methods:**

Using the Gene Expression Omnibus (GEO) profiles of the National Center for Biotechnology Information, the associations between mRNA expression of biomarkers and sarcopenia were explored, including high temperature requirement serine protease A1 (HtrA1), procollagen type III N-terminal peptide (P3NP), apelin, and heat shock proteins 70 (Hsp72). We enrolled 408 community-dwelling adults aged 65 years and older with sarcopenia and nonsarcopenia based on the algorithm proposed by the Asian Working Group for Sarcopenia (AWGS). Muscle strength is identified by hand grip strength using an analogue isometric dynamometer. Muscle mass is estimated by skeletal mass index (SMI) using a bioelectrical impedance analysis. Physical performance is measured by gait speed using 6 m walking distance. The associations between these biomarkers and sarcopenia were determined using receiver operating characteristic (ROC) curve analysis and multivariate regression models.

**Results:**

From the GEO profiles, the sarcopenia gene set variation analysis score was correlated significantly with the mRNA expression of APLNR (*p* < 0.001) and HSPA2 (*p* < 0.001). In our study, apelin was significantly associated with decreased hand grip strength with β values of − 0.137 (95%CI: − 0.229, − 0.046) in men. P3NP and HtrA1 were significantly associated with increased SMI with β values of 0.081 (95%CI: 0.010, 0.153) and 0.005 (95%CI: 0.001, 0.009) in men, respectively. Apelin and HtrA1 were inversely associated with the presence of sarcopenia with an OR of 0.543 (95%CI: 0.397–0.743) and 0.003 (95%CI: 0.001–0.890) after full adjustment. The cutoff point of HtrA1 was associated with the presence of sarcopenia with an OR of 0.254 (95%CI: 0.083–0.778) in men. The cutoff point of apelin was negatively associated with the presence of sarcopenia with an OR of 0.254 (95%CI: 0.083–0.778).

**Conclusion:**

Our study highlights that P3NP, HtrA, and apelin are useful for diagnosis of sarcopenia in the clinical setting.

## Introduction

The term “sarcopenia” was first proposed in 1989 to describe the age-related decline of muscle mass that affects mobility, nutritional status, and independence [1]. The operational definition for sarcopenia proposed by European Working Group on Sarcopenia in Older People (EWGSOP) in 2010 has become the most widely used in the world which both muscle quantity and quality were cardinal requirements [2]. In 2019, Asian Working Group for Sarcopenia (AWGS) proposed an updated diagnostic algorithm based on Asian data, which sarcopenia is defined as low skeletal muscle mass with low muscle strength or low physical performance [3]. Sarcopenia is suggested to be a progressive and degenerative loss of skeletal muscle mass and muscle strength that occurs with aging that is associated with increased adverse outcomes [4]. Since its original description, the concept of sarcopenia has become more complicated and now involves multifactorial pathogenesis. The pathophysiology mechanisms of sarcopenia includes endocrine dysfunctions, neuromuscular junction, growth factors, inflammatory conditions, and muscle protein turnover [5, 6]. Based on these pathways, several biomarkers were proposed to be used for detecting and identifying the development of sarcopenia [7, 8].

However, the diagnosis of sarcopenia depends only on clinical, functional, and imaging parameters valuated after disease onset. It is essential to apply molecular biomarkers in the early diagnosis and prognosis of sarcopenia [9]. A previous study have reported several biomarkers such as high temperature requirement serine protease A1 (HtrA1), procollagen III N-terminal peptide (P3NP), apelin, and heat shock protein 70 (Hsp72) are possibly involved in different components of sarcopenia [10]. The aim of this study is to identify the role of potential biomarkers in the early diagnosis of sarcopenia in a Taiwanese older adult population.

To estimate the sample size of the study, we originally admitt a degree of error in the estimate of 5% and a confidence interval of 95% and have 80% power. Based on the study proposed by Asian Working Group for Sarcopenia (AWGS), the prevalence of sarcopenia in Asian population is about 12.9–15.9 %[11]. It was calculated as the minimum sample size necessary to include at least 173–206 participants to obtain an acceptable estimate. Therefore, our sample size exceeds 200 cases that allow us to make a statistically reliable sample of the prevalence of sarcopenia.

## Method

### Study design and recruitment of participants

**Fig. 1:**
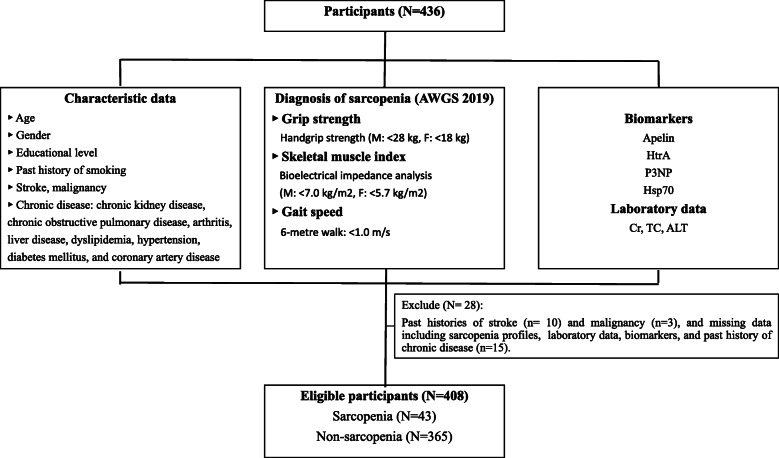
Flow chart of the study design

In this cross-sectional study, the population was obtained during routine health checkup at Tri-Service General Hospital (TSGH), Taiwan, during 2015–2018. According to the flow chart shown in Fig. 1, 436 people aged 65 years or more were included in the study. A series of examination was performed as follows: clinical measures of sarcopenia included muscle strength, skeletal muscle mass, and gait speed were collected. Baseline laboratory data and biomarkers, including P3NP, HtrA1, apelin, and Hsp72 were collected from the participants. These participants were asked whether they had smoking history and medical history of stroke, malignancy, and chronic disease such as chronic kidney disease, chronic obstructive pulmonary disease, arthritis, liver disease, dyslipidemia, hypertension, diabetes mellitus, and coronary artery disease. Exclusion criteria included those with history of stroke (*n* = 10), malignancy (*n* = 3), or missing data included sarcopenia profiles, laboratory data, biomarkers, and past history of chronic disease. Finally, 43 participants with sarcopenia and 365 participants without sarcopenia were included in the subsequent analysis. All procedures were approved by the Institutional Review Board of TSGH (No. 2–103–05-024) in accordance with the revised Helsinki Declaration. We have obtained patient permission before enrollment by asking them to complete a wriiten informed consent.

### Gene expression analysis from the gene expression omnibus (GEO) profiles

Gene expression of APLNR (apelin), HTRA1 (HtrA1), COL3A1 (P3NP) and HSPA2 (Hsp72) was obtained from the NCBI GEO database (accession ID: GSE18732) [12]. This dataset contains various physiological measurements and whole gene expression data of skeletal muscle (salverus lateralis) from 118 participants. Clinical information was selected to include demographic characteristics, medication, laboratory data, and fat mass and lean mass in the arms, legs, and trunk. To assess the extent of Sarcopenia by gene expression, we used the Sarcopenia gene set downloaded from the Enrichr database [13], which includes 27 genes that have been linked to sarcopenia in various literatures. To obtain individual Sarcopenia scores, we used the Gene Set Variation Analysis (GSVA) scoring strategy with default parameter settings for the analysis [14]. The analysis was performed in R using GSVA package (Version 1.38.2) according to the reference manual, and the enrichment score (ES) of the Sarcopenia gene set was calculated for each subject. A high GSVA score represents an overall higher expression of the relevant genes in the sample, therefore reflects the degree of activation of the sarcopenia gene set in the sample.

### Diagnosis of sarcopenia

According to the algorithm proposed by the AWGS in 201 9[3], possible sarcopenia is determined when low muscle strength with or without reduced physical performance occurs. A diagnosis of sarcopenia is defined when reduced skeletal muscle mass appears with low muscle strength or low physical performance. When all criteria are displayed, sarcopenia is considered severe. In our study, muscle strength is identified by hand grip strength using an analogue isometric dynamometer (North Coast Hydraulic Hand Dynamometer, North Coast Medical Inc., Morgan Hill, CA). Muscle mass is estimated by skeletal mass index (SMI) using a bioelectrical impedance analysis (BIA) (InBody720, Biospace, Inc., Cerritos, CA, USA). SMI is calculated by dividing the skeletal muscle mass (kg) by the square of the height (m^2^). Physical performance is measured by gait speed using six meters walk. The cutoff points of hand grip strength are 28.0 kg for men and 18.0 kg for women. The cutoff points of SMI measuring by BIA are 7.0 kg/m2 for men and 5.7 kg/m2 for women. The cutoff point of gait speed are 1.0 m/s for both [2].

### Biomarkers

We examined these multidimensional biomarkers to determine the underlying pathophysiological mechanisms, including P3NP, HtrA1, apelin, and Hsp72. According to the manufacturer’s instructions, plasma P3NP (Wuhan Fine Biotech Co., Ltd., Wuhan. China), HtrA1 (Cloud-Clone Corp., Houston, TX, USA), apelin (Phoenix Pharmaceuticals, Belmont, CA, USA, and Hsp72 (Enzo Life Science Inc., Farmingdale, NY, USA) were performed using a competitive enzyme-linked immunoassay (ELISA). The lower detection limit was 156 pg/mL for P3NP, 31.2 pg/mL for HtrA1, 70 pg/mL for apelin and 200 pg/mL for Hsp72. During the sample analysis period, the variation of these assays ranged from 5 to 10%.

### Study variables

Educational level, past history of cigarette smoking, and chronic disease included chronic kidney disease, chronic obstructive pulmonary disease, arthritis, liver disease, dyslipidemia, hypertension, diabetes mellitus, and coronary artery disease was obtained from the self-reported questionnaires. Laboratory data included creatinine (Cr), total cholesterol (TC), and alanine aminotransferase (ALT) were collected from blood samples using standard procedures.

### Statistical analysis

For all statistical analyses, we used the Statistical Package for the Social Sciences, version 22.0 (SPSS Inc., Chicago, IL, USA), for Windows. The normal distribution of data and variables were checked by Kolmogorov–Smirnov normality test. We stratify all participants in to male and female group due to significant findings of the interaction testing. In order to compare nonparametric variables in the test groups, we used Pearson chi-square test for qualitative variables and the Mann-Whitney U test for two independent groups to estimate the distributions. Spearman’s R correlation test was used to evaluate the relationships between the analyzed variables. The threshold for statistical significance was defined as *p*-value lower than 0.05. Correlations among sarcopenia GSVA scoring and arm, leg, and trunk fat mass and lean mass were determined using Pearson’s correlation coefficient. We analyzed associations between biomarkers and sarcopenia parameters using a linear regression model. Natural Log transformation was used to normalize the distributions of the biomarkers. To adjust these relationships, we used the variables, including age, education, number of chronic disease, and history of cigarette smoking. In addition, explained variance (Nagelkerke R^2^) was used to select the optimal model for the biomarkers. We used a receiving operating characteristic (ROC) analysis to determine the cutoff points of biomarkers as well as the area under the ROC (AUROC). Logistic regression model was used to determine the relationships between the cutoff points of the biomarkers and the odds ratio (OR) of the occurrence of sarcopenia.

## Result

### Association of the sarcopenia GSVA score with the mRNA expression of APLNR, HTRA1, COL3A1, and HSPA2

**Fig. 2:**
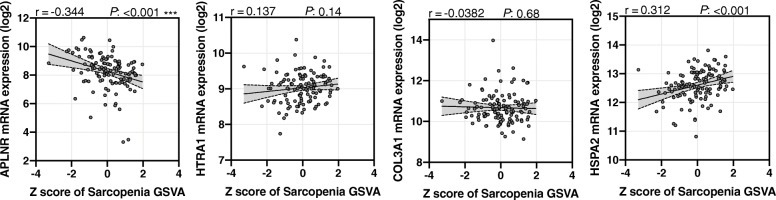
Relationship between the several biomarkers and sarcopenia GSVA scoring via the DisGeNET databases

To determine which biomarkers were significantly correlated with the GSVA score of sarcopenia, we evaluated the relationships between several biomarkers and sarcopenia GSVA scoring via the DisGeNET databases (Fig. 2). Figure 2 shows that the sarcopenia GSVA score was significantly correlated with the mRNA expression of APLNR (*p* < 0.001) and HSPA2 (*p* < 0.001), but not with HTRA1 and COL3A1 mRNA expression. The lean mass of the arm, leg, and limb were not significantly negatively correlated with the mRNA expression of APLNR, HTRA1, COL3A1, and HSPA2.

### Study population characteristics

Table 1 displays the baseline population and clinical variables of participants with and without sarcopenia. The mean ages of sarcopenia and control group were 76.92 ± 8.68 and 74.01 ± 37.63, respectively. Sarcopenia group had significantly higher proportion of smoking history than non-sarcopenia group. Participants without sarcopenia had significantly greater hand grip strength, SMI, and gait speed than those with sarcopenia. In terms of the biomarkers included in the study, sarcopenia group had significantly lower HtrA1 and apelin levels than control group, but no significant differences were found in other biomarkers.

**Table 1:** Characteristics of Study Population

Variables	Sarcopenia (***N*** = 43)	Non-sarcopenia (***N*** = 365)	***P***-value
**Continuous variables, (SD)**			
**Age (years)**	76.92 (8.68)	74.01 (37.63)	0.166
**Grip strength (kg)**	18.88 (6.12)	25.31 (8.85)	< 0.001
**SMI (kg/m**^**2**^**)**	5.81 (0.82)	6.87 (1.01)	< 0.001
**Gait speed (m/s)**	1.04 (0.36)	1.25 (0.32)	< 0.001
**P3NP (mg/L)**	0.53 (1.79)	0.41 (1.56)	0.661
**HtrA1 (ug/L)**	0.09 (0.04)	0.11 (0.06)	0.008
**Apelin (ng/mL)**	1.08 (0.66)	4.49 (15.81)	< 0.001
**Hsp72 (ng/mL)**	2.72 (4.21)	2.24 (4.45)	0.477
**Cr (mg/dL)**	0.84 (0.30)	0.86 (0.25)	0.743
**TC (mg/dL)**	186.16 (38.14)	183.04 (34.76)	0.591
**ALT (U/L)**	17.98 (9.43)	18.75 (10.75)	0.608
**Categorical variables (%)**			
**Gender (male) (%)**	32 (62.7)	201 (40.1)	0.002
**Smoking (%)**	49 (96.1)	402 (83.1)	0.014
**Education (high school) (%)**	26 (52.0)	317 (65.5)	0.058
**Number of chronic disease**	1.28 (0.99)	1.01 (1.01)	0.075

*SMI* Skeletal muscle index, *Cr* Creatinine, *TC* Total cholesterol, *ALT* Alanine aminotransferase, *P3NP* Procollagen-3 N-Terminal Peptide, *HtrA1* High temperature requirment serine protease A1, *Hsp72* Heat shock proteins 70

### Association between biomarkers and sarcopenia in gender difference

Table 2 analyzes the associations between P3NP, HtrA1, apelin, and Hsp72 and sarcopenia components, including grip strength, SMI, and gait speed, in both men and women. After full adjustment, apelin was significantly associated with decreased hand grip strength with β values of − 0.137 (95%CI: − 0.229, − 0.046) in men. P3NP and HtrA1 were significantly associated with increased SMI with β values of 0.081 (95%CI: 0.010, 0.153) and 0.005 (95%CI: 0.001, 0.009) in men, respectively. However, no significant difference was detected in the relationship between biomarkers and gait speed.

**Table 2:** Association between different biomarkers and sarcopenia components in gender difference

		Unadjusted β (95% CI)	R^**2**^	***P*** Value	Fully adjusted β (95% CI)	R^**2**^	***P*** Value
**Variable**		**Outcome variable: Hand grip strength (as a continuous variable)**					
**Male**	**P3NP**	0.089 (− 0.504, 0.682)	0.001	0.767	0.217 (− 0.320, 0.753)	0.215	0.426
	**HtrA1**	0.011 (−0.014, 0.037)	0.005	0.386	0.006 (− 0.018, 0.030)	0.172	0.627
	**Apelin**	−0.101 (− 0.203, 0.001)	0.023	0.052	− 0.137 (− 0.229, − 0.046)	0.250	0.003
	**Hsp72**	− 0.113 (− 0.372, 0.147)	0.004	0.393	0.018 (− 0.222, 0.258)	0.205	0.883
**Female**	**P3NP**	−0.239 (− 0.907, 0.429)	0.002	0.482	−0.326 (− 0.927, 0.274)	0.214	0.285
	**HtrA1**	0.001 (−0.014, 0.017)	0.001	0.851	0.004 (−0.010, 0.018)	0.240	0.562
	**Apelin**	0.019 (−0.026, 0.064)	0.003	0.409	0.010 (−0.031, 0.051)	0.198	0.638
	**Hsp72**	−0.007 (− 0.190, 0.175)	0.001	0.937	−0.102 (− 0.267, 0.064)	0.222	0.227
		**Outcome variable: Skeletal muscle index (as a continuous variable)**					
**Male**	**P3NP**	0.079 (0.004, 0.154)	0.038	0.040	0.081 (0.010, 0.153)	0.186	0.026
	**HtrA1**	0.005 (0.001, 0.009)	0.054	0.024	0.005 (0.001, 0.009)	0.122	0.030
	**Apelin**	0.096 (−0.035, 0.228)	0.019	0.148	0.090 (−0.040, 0.220)	0.119	0.173
	**Hsp72**	0.004 (−0.032, 0.039)	0.001	0.842	0.004 (−0.030, 0.038)	0.123	0.834
**Female**	**P3NP**	−0.017 (− 0.119, 0.085)	0.001	0.743	−0.016 (− 0.117, 0.085)	0.055	0.752
	**HtrA1**	−0.001 (− 0.005, 0.002)	0.005	0.441	−0.001 (− 0.005, 0.003)	0.050	0.554
	**Apelin**	0.008 (−0.087, 0.103)	0.001	0.865	0.004 (−0.091, 0.099)	0.055	0.934
	**Hsp72**	0.015 (−0.014, 0.043)	0.007	0.319	0.007 (−0.022, 0.037)	0.051	0.637
		**Outcome variable: Gait speed (as a continuous variable)**					
**Male**	**P3NP**	0.021 (−0.007, 0.048)	0.019	0.142	0.022 (−0.004, 0.049)	0.172	0.093
	**HtrA1**	0.001 (−0.001, 0.002)	0.013	0.273	0.001 (−0.001, 0.002)	0.210	0.357
	**Apelin**	0.032 (−0.016, 0.079)	0.016	0.191	0.017 (−0.029, 0.063)	0.156	0.468
	**Hsp72**	−0.012 (− 0.025, 0.001)	0.030	0.062	−0.012 (− 0.024, 0.001)	0.160	0.059
**Female**	**P3NP**	0.032 (−0.009, 0.073)	0.017	0.123	0.025 (−0.009, 0.060)	0.335	0.143
	**HtrA1**	0.001 (−0.002, 0.001)	0.003	0.551	0.001 (−0.001, 0.001)	0.289	0.963
	**Apelin**	−0.010 (− 0.047, 0.026)	0.002	0.572	−0.016 (− 0.048, 0.015)	0.290	0.315
	**Hsp72**	0.001 (−0.011, 0.012)	0.001	0.932	−0.004 (− 0.014, 0.006)	0.295	0.414

Fully adjusted: adjustment for age, education, smoking history, number of chronic disease

### Association between biomarkers and the presence of sarcopenia

In Table 3, apelin was associated with dcreased presence of sarcopenia with an OR of 0.543 (95%CI: 0.397–0.743) after full adjustment. HtrA1 was associated with decreased presence of sarcopenia with an OR of 0.003 (95%CI: 0.001–0.890) after full adjustment. Nevertheless, no significant association was noted in other biomarkers.

**Table 3:** Association between biomarkers and the presence of sarcopenia

Biomarkers	***Sarcopenia***			
	Unadjusted OR (95% CI)	***P***-Value	Fully adjusted OR (95% CI)	***P***-Value
**Apelin**	0.511 (0.374–0.698)	< 0.001	0.543 (0.397–0.743)	< 0.001
**P3NP**	1.046 (0.873–1.254)	0.623	1.051 (0.871–1.267)	0.607
**HtrA1**	0.002 (0.001–0.511)	0.032	0.003 (0.001–0.890)	0.047
**Hsp72**	1.027 (0.963–1.096)	0.415	1.015 (0.949–1.086)	0.664

Fully adjusted: age, education, smoking history, number of chronic disease

### Optimal cutoff points in P3NP, HtrA1, apelin, and Hsp72

Based on the results mentioned above, we further calculated the optimal cutoff points of P3NP, HtrA1, apelin, and Hsp72 using ROC curve analysis to identify the ability of these biomarkers to detect the presence of sarcopenia (Table 4). The AUROC was 0.578 (95%CI: 0.482–0.675; sensitivity: 48.8%, specificity: 66.5%), 0.593 (95%CI: 0.506–0.679; sensitivity: 38.0%, specificity: 89.5%), 0.709 (95%CI: 0.651–0.767; sensitivity: 47.3%, specificity: 95.8%), and 0.606 (95%CI: 0.524–0.688; sensitivity: 75.6%, specificity: 49.0%) in P3NP, HtrA1, apelin, and Hsp72, respectively. The cutoff point was determined by Youdan index that P3NP was 0.045 in men and 0.035 in women; HtrA1 was 0.067 in men and 0.035 in women; apelin was 0.911 in men and 0.863 in women; Hsp72 was 0.135 in men and 0.150 in women.

**Table 4:** Optimal cut-off points in P3NP, HtrA1, Hsp72, and Apelin

	P3NP	HtrA1	Apelin	Hsp72
**AUC (95%CI)**	0.578 (0.482–0.675)	0.593 (0.506–0.679)	0.709 (0.651–0.767)	0.606 (0.524–0.688)
**Sensitivity**	48.8%	38.0%	47.3%	75.6%
**Specificity**	66.5%	89.5%	95.8%	49.0%
***P*-value**	0.093	0.063	< 0.001	0.020
**Cutoff points in men**	0.045	0.067	0.911	0.135
**Cutoff points in women**	0.035	0.035	0.863	0.150

### Association between cutoff points of biomarkers and the presence of sarcopenia in gender difference

In Table 5, HtrA1 was associated with the presence of sarcopenia with an OR of 0.254 (95%CI: 0.083–0.778) in men after full adjustment. Apelin was significantly associated with the presence of sarcopenia with an OR of 0.254 (95%CI: 0.083–0.778). In addition, apelin was negatively associated with the presence of sarcopenia with an OR of 0.283 (95%CI: 0.123–0.655) without adjustment in men, however, the *p*-value was slightly elevated after full adjustment that no significant difference was noted in the association with the presence of sarcopenia.

**Table 5:** Gender difference in Association between cutoff points of biomarkers and the presence of sarcopenia

Gender	Biomarkers	Cutoff points	Sarcopenia			
			Unadjusted OR (95% CI)	***P***-Value	Fully adjusted OR (95% CI)	***P*** -Value
**Male**	**Apelin**	0.911	0.283 (0.123–0.655)	0.003	0.433 (0.176–1.064)	0.068
	**P3NP**	0.045	1.960 (0.855–4.495)	0.112	1.043 (0.988–1.100)	0.126
	**HtrA1**	0.067	0.306 (0.113–0.833)	0.020	0.254 (0.083–0.778)	0.016
	**Hsp72**	0.135	3.373 (1.292–8.811)	0.013	2.350 (0.854–6.464)	0.098
**Female**	**Apelin**	0.863	0.324 (0.122–0.864)	0.024	0.360 (0.131–0.989)	0.048
	**P3NP**	0.035	3.777 (1.023–13.940)	0.046	1.059 (0.975–1.150)	0.172
	**HtrA1**	0.036	0.331 (0.064–1.724)	0.189	0.305 (0.053–1.769)	0.186
	**Hsp72**	0.150	2.244 (0.753–6.691)	0.147	2.216 (0.725–6.776)	0.163

Fully adjusted: age, education, smoking history, number of chronic disease

## Discussion

Diverse pathophysiological mechanisms contribute to the process of sarcopenia. It is imperative to identify individuals at risk of sarcopenia and prevent the progression to disability and functional dependence, or even medical institutionalization of the older adult population. In the present study, we investigated the relationships between biomarkers and sarcopenia among Taiwanese older adults. We found that HtrA1 was associated with decreased presence of sarcopenia in older men and apelin is significantly associated with reduced occurrence of sarcopenia, in women. Our findings suggest that molecular biomarkers may represent a beneficial effect on aging and an essential tool for early diagnosis of sarcopenia in older adult population.

Apelin is a novel adipokine that is expressed in different tissues and secreted by.

both human and mouse adipocytes [15–17]. It is believed that apelin can be used to reduce insulin resistance with the aim of improving altered glucose metabolism [18]. Vinel et al. demonstrated that apelin synthesis in skeletal muscle is reduced and plasma apelin levels decrease in aged mouse [19]. Conversely, after supplementation with apelin injection per day, improved skeletal muscle capacities and myofiber hypertrophy were noted. Several researchers have considered apelin as an adipokine because of its upregulation in adiposity mass with obesity and improvement of cardiometabolic diseases [20–22]. This mediator has a beneficial effect on energy metabolism by increasing insulin sensitivity and glucose uptake [23]. Emerging research has indicated Apelin as a myokine from skeletal muscle that might have an influence on muscle physiology and function [24]. Apelin is suggested to induce the promotion of protein synthesis and inhibition of age-related proteolysis in aged myotubes [19]. On the other hand, apelin can trigger mechanisms of myogenesis and angiogenesis coupling that leads to an improvement in regenerative processes in the muscle cells of aged mice [25]. Apelin has been found as an independent predictor of bone mineral density in postmenopausal women [26]. It has an inhibitory effect on gonadotropin and prolactin secretion in females [27]. We speculated that the relationship between apelin and sarcopenia might be the interaction after menopause.

HtrA1, a protease of the family [28], is regarded as an important role in the inflammation via transforming growth factor-β (TGF-β) inhibition [29]. HtrA1 is involved in the pathophysiological mechanisms of these diseases, such as osteoarthritis, dementia, and age-related macular degeneration [30, 31]. Lorenzi et al. first described the association between HtrA1 and frailty in older population [32]. Loss of HtrA1 was reported to impair muscle cell maturation via TGF-β pathway [33]. Emerging study proposed that HtrA protease deficiency induced denervation-independent skeletal muscle degeneration with sarcopenia [34]. In our study, we found that HtrA1 was associated with the presence of sarcopenia, particularly in men. However, rare studies discussed the connection of HtrA1 and gender difference. The potential role of this biomarker warrants further longitudinal studies to explore in the association with sarcopenia.

Hormones, including testosterone, growth hormone, and dehydroepiandrosterone are potential biomarkers of sarcopenia [35–37]. Age-related reductions in protein synthesis and muscle function have been linked to age-related declines in sex hormones and growth factors [38]. Testosterone can increase plasma P3NP levels in a dose-dependent manner and stimulate the expression of several skeletal muscle transcripts and proteins [39]. Changes in levels of P3NP have been regarded as an early marker of muscle quality and function. In males, high levels of P3NP have been noted when the normal increases in skeletal mass in response to endurance-type exercise [40]. In a clinical trial of 106 elderly men, increases in P3NP induced by growth hormone treatment were found to be related to greater gains in total and appendicular skeletal mass [41]. The exercise intervention is believed to increase circulating P3NP and is positively correlated with changes in muscle mass [42]. Collectively, our findings are consistent with the results of these studies, however, future studies should examine other mechanisms as well, especially the role of apelin and P3NP in skeletal muscle function.

This study had some limitations that should be acknowledged. The major limitation is that we can’t identify the causal relationship between biomarkers and sarcopenia because of the study design. It is essential to conduct longitudinal studies to examine whether molecular biomarkers applying for early diagnosis and to evaluate clinical intervention in sarcopenia. Second, all participants included in this study were non-institutionalized and relatively healthy. It might lead to the underestimation of the prevalence of sarcopenia. Next, some potential confounding factors influencing sarcopenia, including physical activity, dementia status, nutritional status, number of medications, and body mass index were not included in the analysis, because these variables were not recorded in the health checkup. Last, we included only Taiwanese participants in this study. Thus, the generalization of the results to other population may be precluded.

## Conclusion

In the present study, we found that HtrA1 and apelin were significantly associated with sarcopenia and provide a potential approach in molecular diagnosis of in older adult population. Our findings would help us more recognize the pathophysiologic mechanisms of this disorder. Sarcopenia is a multifactorial pathogenesis condition and is considered to be highly prevalent and carry a high risk for adverse health outcomes. It is important to determine a clear definition and diagnostic criteria of sarcopenia to guide both clinical practice and research design.

## Data Availability

The datasets generated and analyses performed during the current study are not publicly available due to the consent requirement of participants, but sex and age decade-stratified descriptive data are available from the corresponding author on reasonable request.

## Abbreviations

EWGSOP: European Working Group on Sarcopenia in Older People
AWGS: Asian Working Group for Sarcopenia
TSGH: Tri-Service General Hospital
P3NP: Procollagen type III N-terminal peptide
HtrA1: High temperature requirement serine protease A1
Hsp72: Heat shock proteins 70
GEO: Gene Expression Omnibus
SMI: Skeletal mass index
Cr: Creatinine
TC: Total cholesterol
ALT: Alanine aminotransferase
ROC: Receiving operating characteristic
CI: Confidence interval
OR: Odds ratio
IRB: Institutional Review Board
GSVA: Gene Set Variation Analysis
ELISA: Enzyme-linked immunoassay
TGF-β: Transforming growth factor-β
