# Correction to: Cross-sectional associations among P3NP, HtrA, Hsp70, Apelin and sarcopenia in Taiwanese population

**DOI:** 10.1186/s12877-022-02882-2

**Published:** 2022-04-20

**Authors:** Yuan-Yuei Chen, Yi-Lin Chiu, Tung-Wei Kao, Tao-Chun Peng, Hui-Fang Yang, Wei-Liang Chen

**Affiliations:** 1 Department of Pathology, Tri-Service General Hospital, and School of Medicine, National Defense Medical Center, Taipei, Taiwan, Republic of China; 2 Department of Pathology, Tri-Service General Hospital Songshan Branch; and School of Medicine, National Defense Medical Center, Taipei, Taiwan, Republic of China; 3 Division of Geriatric Medicine, Department of Family and Community Medicine, Tri-Service General Hospital, and School of Medicine, National Defense Medical Center, Number 325, Section 2, Chang-gong Rd, Nei-Hu District, 114 Taipei, Taiwan, Republic of China; 4 Department of Biochemistry, National Defense Medical Center, Taipei, Taiwan, Republic of China

**Correction to: BMC Geriatr 21, 1-9 (2021)**

**https://doi.org/10.1186/s12877-021-02146-5**

After publication of this article [1], the authors reported that the data in Table 1, lines "Gender", "Smoking" and "Education" were incorrect.

The original article [1] has been updated.
